# The physical foundation beneath protein generative modeling

**Published:** 2026-09-03

**Authors:** Tianyu Lu, Po-Ssu Huang

**Affiliations:** a Department of Bioengineering, Stanford University, Stanford, California, USA

## Abstract

Physical equations in protein modeling appear to have been replaced by generative models trained directly on structure data. By learning a mapping from noise to data, sampling *de novo* protein structures has become much more efficient. However, such models can also learn non-physical features and break down with out-of-distribution settings which are typical in protein design campaigns. In this review, we highlight where the physics persists in protein generative modeling pipelines and the issues that linger when physically relevant components of a macromolecular system are left unmodeled. We present a perspective that respecting the underlying physics of macromolecular systems, increasingly through learned representations that are physically grounded and updating generative models with experimental data, is foundational to generative modeling for functional protein design.

## 1. Introduction

The modeling of macromolecules has historically required physical equations: force fields, energy functions, rotamer libraries, and molecular-mechanics terms [1, 2, 3]. The Rosetta suite of tools, at its core, is the Rosetta energy function: van der Waals packing, hydrogen bonding, electrostatics, implicit solvation, and data-driven terms from the Protein Data Bank (PDB) [3]. The terms of the energy function were partially tuned to distinguish decoys, i.e. synthetic structures, from crystallographic structures. Recently, however, the field has shifted away from physical equations towards generative models trained on structural data [4, 5, 6, 7]. One distinguishing advantage is their ability to sample from the distribution of structures much more efficiently than the steps required using physics-derived methods. Another more critical feature is the ability to implicitly capture relations in the data that resemble physical interactions by learning. Neural network weights can acquire information correlated with physical equations when fitted to the data distribution. In presenting macromolecular data to the network, the information can be grounded in physical inputs such as coordinates and bond geometries, but can also be non-physical inputs which could lead the model to learn non-physical correlations. In this review, we discuss the issues that arise when physics is not modeled and highlight the current generative modeling paradigm where physics-based modeling remains important.

## 2. Generative models as physical priors

Generative models learn a distribution of its training data. By training on experimentally determined protein structures, the physics which governs physically plausible structures are implicitly modeled. The probability mass defined in the model is concentrated where folded proteins have been observed, and a well-trained model produces samples drawn from that region without evaluating any explicit energy. The distribution it approximates is the empirical distribution of deposited structures, which are dominated by proteins that fold, crystallize, and are often cryo-cooled, which is related but not equivalent to the distribution of structures in solution or in their native cellular environments at relevant temperatures, pH, and solvents. Such models serve as priors for protein design tasks which seek to draw samples from a bespoke region of the total space of physically plausible structures [8]. Many design tasks necessitate the proposal of a novel sequence or structure to achieve an objective previously not observed in the training data. The proposed sample therefore resides, to some degree, outside the training data distribution, and the success of a design campaign depends on the generalizability of the models used [9].

## 3. Steering the prior towards a design objective

### 3.1. Physical terms as filters and guidance

In design, the prior distribution learned by a generative model is almost always different from the distribution from which we wish to sample. For example, the distribution of all protein-protein interfaces is different from the distribution of high affinity interfaces with minimal off-target binding. This distribution shift can be approximated by *post hoc* filters. BindCraft applies Rosetta-based physical terms [10] to filter generated designs to ground them in physical plausibility, together with confidence metrics from black-box structure prediction based oracles [11], to enrich samples that are likely to be high affinity binders. However, structure prediction models are often only trained on positive data. In the binder design setting, confidence metrics capture the confidence of the predicted pose, implicitly conditional on the assumption that the interaction is real. *Post hoc* filters are bounded by what the generator already proposes and inherits the generalizability limits of the oracles. A meta-analysis of 3,766 experimentally characterized binders show that physics-based metrics provide additional predictive power to sort *in silico* binders from non-binders compared to structure prediction metrics alone, such as ipSAE [12]. Physical terms can also be applied during the generation process of the sample by steering the denoising process with Feynman-Kac resampling, beam search, or reinforcement learning against non-differentiable physical validity rewards [7, 13, 14]. Physical plausibility from the perspective of a predictive model could also be transferred to generative models by finetuning predictive models as generative models, such as RFdiffusion which builds on RoseTTAFold [15, 16]. Whether using physical terms as filters or guidance, their intended effect is to shift the prior learned by the generative model towards a more bespoke distribution for a given design objective (Figure 1).

#### Limitations of structure prediction models for ranking designs

Using folding models as oracles may have unintended consequences. For example, charge inversion mutations, which were expected to electrostatically abolish an interaction with the negatively charged ATP, did not perturb the predicted ligand binding pose nor substantially reduce the confidence [17]. These adversarial examples suggest that co-folding models tend to interpolate within its training data distribution and do not adequately capture physical expectation [18, 19]. Additionally, use of prediction-derived metrics such as ipSAE often results in ranking false positive designs highly, according to the general distribution of interfaces, while failing to distinguish subtle incompatibilities within the samples (Figure 2). These issues highlight the so-called activity-cliff or rugged landscape of proteins, where small changes to the sequence or structure lead to dramatic changes in its physical plausibility or function. A strategy to circumvent this is to introduce negatives during the inference time of generative models. In autoguidance, a generative model is guided with a “bad” version of itself, typically an under-trained model which produces samples of poor quality, such that sampling is pushed away from the failure modes of the under-trained model [20]. This approach is similar in motivation to tuning the Rosetta energy function to separate decoys from natives, but has the advantage of being more flexible and is not subject to the expressibility constraints imposed by physical equations with much fewer parameters. A similar behavior occurs on the generalization of both design models and predictive models on longer length proteins. While subject to the same physics, longer proteins tend to underperform in designability metrics [8, 21]. The struggle with length generalization points to the same phenomenon, that physics is not adequately captured by generative models or by structure prediction oracles, since length is a statistical property of the training set rather than a physical variable.

#### Grounding models in physics-based representations and architectures

##### Using physical energies to organize an all-atom latent space

A recent class of models attempts to focus the network on relevant physical information by redefining the representations of molecules to more fundamental building blocks, typically a collection of atoms within a few Ångstroms of range [22, 23]. Efforts in machine learning interaction potential (MLIP) development have directed their learning systems towards predicting inter-atomic energy by fitting models to pre-computed classical or quantum calculations [24, 25], with the goal of speeding up traditionally slow and expensive calculations. The emphasis on local interactions is also critical for model generalizability. Models that only use structure-based training objectives can readily complete structural reconstruction tasks by memorizing non-generalizable features of a protein. In contrast, leveraging information from physically-grounded features is likely to be more generalizable to unseen problems. However, there is an informational gap between the localized atomic features and the global fold of a protein. A model that directly bridges this gap could overcome the performance cliffs observed due to memorization of non-physical features by assessing its ability to use strictly local features to reconstruct the global fold. Recent work showing the convergence of internal representations of scientific foundation models hint at a common physical structure learned by otherwise dissimilar models [26, 27]. To disentangle the bottom-up physical features and the context associated with the global structure, SLAE leverages an asymmetrical encoder-decoder architecture. It attempts to project global structure to a latent space by using only localized information to encode it, while simultaneously trying to achieve accurate reconstruction with a transformer-based decoder. Ablation analyses showed that predicting energy by the decoder was essential during model training for the localized all-atom representation to perform well simultaneously at global fold reconstruction and at downstream tasks such as NMR chemical shift prediction, function prediction, and all-atom sidechain reconstruction. Remarkably, the model can achieve physically accurate and smooth linear interpolation between protein states. This hints at a well-organized latent space: local all-atom representations now need to be organized along physically meaningful axes by virtue of the asymmetric decoder’s energy supervision task, jointly with global structure reconstruction. This prevents memorization of spurious features of the training data. This approach can be viewed as a hybrid between purely data-driven representations and closed-form energy functions, inheriting both the generalizability from the physical supervision and the expressivity of representation learning. A similar approach is the affinity module in Boltz-2 where the organization and information content of the denoising module representations are updated during training to better predict binding likelihoods and affinities [28].

##### Physics-inspired architectural regularization

Physical models also inspire architectures of protein design models. The Potts model, originally developed for modeling interacting spins on a crystalline lattice, has had successful applications in protein design [29]. Earlier methods rely on co-evolutionary information derived from multiple sequence alignments to infer the Potts model parameters. Recently, models such as TERMinator, ChromaDesign, and Caliby parameterize the Potts model with outputs of a neural network [6, 30, 31]. The decision to use a Potts model to parameterize the distribution of sequences from which to sample may seem counterintuitive given the flexibility of autoregressive models, as the latter can capture any arbitrary distribution over sequences, including higher-order correlations beyond second order. However, several works indicate that second order terms are necessary and sufficient to capture protein structure and function [32, 33, 34]. Thus restricting to only first and second order terms in a Potts model can be viewed as a form of physics-inspired architectural regularization. It serves as a parameterization bottleneck to prevent the modeling of possibly spurious higher order terms, which may explain the drop in designability for ProteinMPNN compared to ChromaDesign and Caliby, especially for longer protein lengths [31].

#### Connecting generative model hypotheses with experiment

##### Distilling the necessary and sufficient physical conditions for functional protein design

Achieving successful “zero-shot” design highlights the successful distillation of physical principles into necessary and sufficient conditioning inputs to modern protein design tools. A *de novo* designed protein was able to bind the drug exatecan which has no prior structure in the PDB nor the Cambridge Structural Database (CSD) by iterating between protein-ligand co-folding with AlphaFold3 and sequence redesign with LASErMPNN, starting from ligand interactions derived from COMBS [35]. A multi-objective genetic algorithm was used to design a *de novo* zinc-dependent phosphatase [36] by iterating between a metal site predictor Metal3D [37] and sequence redesign and repacking with Caliby, starting from a Protpardelle backbone [38]. In a *de novo* serine hydrolase design campaign, PLACER was used to model the preorganization of a DFT-derived active site with scaffolding done by RFdiffusion [15, 39, 40].

##### Implications of unmodeled physical components

As with any modeling approach, there are components of the system that are not modeled, including explicit solvent, energy barriers due to desolvation of the binding pocket, and the entropic versus enthalpic components of binding, among other aspects. Other external constants are also not frequently modeled, including stability at some given pH and temperature, desired protonation states, and competition with off-targets. In many cases, the choice to not model a given component is deliberate, for example hydrogens only being resolved in very high resolution crystal structures. In other cases, the missing components are integral to the success of a design campaign. For example, in a serine hydrolase design campaign, the activated water in the second step of the double-displacement ester hydrolysis reaction is not explicitly modeled while being essential to the enzyme mechanism [39]. In binder design, the often desired selectivity criterion is often not explicitly modeled. With a motivation similar to autoguidance, AlloGen demonstrates that off-target states can be steered against during inference time [41]. Electrostatics form an integral part of physics-based design methods, where accounting for their long-range effect is a challenge for molecular dynamics simulations. However, the modeling of electrostatics is sparse in recent machine learning methods for design and standard MLIPs also do not account for long-range interactions [24]. The orientation of an electric field is thought to be the main contributor to rate acceleration in well-studied enzymes, where contributions to the electric field can come from distal sites of the protein [42, 43, 44]. Charge stabilization by an oxyanion hole is a consequence of this: in the serine hydrolase design campaign, mimicking natural ester hydrolases in constructing the oxyanion hole motif using the residue after the catalytic serine led to a five fold rate improvement [39]. When physical constants are not modeled, fallback to natural occurrence can be a viable strategy [45]. However, modeling electrostatics is dependent on accurate protonation states which exist as an equilibrium and are solvent and pH dependent, all of which are usually not modeled and so are added or predicted with tools decoupled from the generative model, such as applying molecular dynamics *post hoc* to validate rather than steering the generative process [46, 47]. This could explain why low temperature sampling, where models are altered during inference time to concentrate more probability density on its more confident regions, is almost always necessary to produce designable samples, as concentrating samples on the modes of the distribution effectively averages out the unmodeled latent variables in the model.

##### Kinetics as an explicit design objective

Low temperature sampling exacerbates the tendency to sample overly stable designs while not explicitly modeling kinetics, where over-optimization for designability could ignore biologically relevant modes [8]. Most biologically relevant phenomena require a delicate balance between stability and instability [48], all while being constrained by physical plausibility. Broerman *et al*. considered kinetics as an explicit design objective by intentional design of clashes in a strained intermediate which are resolved by dissociation [49]. The dissociation rate can be increased by directing the force of the strained complex to shear along the axis of secondary structure elements, whereas clashes that require rotation to resolve showed slower dissociation rates. In this example, the prudent use of low temperature sampling with RFdiffusion was important to stabilize the fusion of the binder to the conformational switch, since that interaction must be more stable than the desired detaching interface, forcing the dissociation rather than rearrangement of the complex. Nonetheless, the process required manual specification of the strained states not accessible to generative models trained on ground states.

##### Testing designs as plausibility queries in the physical world

A strategy to account for the difficult-to-model components is to include experimental data. This is the classic design-build-test-learn cycle in engineering, for example using experimental data to better fit Rosetta energy terms to reduce overpacking [50]. Generative models serve as priors which can be updated with experimental results using Bayesian optimization [51]. Each design is a hypothesis and queries physical reality, ideally to maximize information gain, typically by maximizing an upper confidence bound of an oracle. In this perspective, the goal of generative model development is to sufficiently map out a hypothesis space that is both easy to sample from and sufficiently expressive such that it is capable of responding to highly nonlinear experimental data. Beyond the baseline assessments of designability, it may be more instructive to evaluate model performance by how efficiently it can adapt its placement of probability density on high quality, but previously out-of-distribution examples. These rounds of iteration can also be entirely lab-driven, with a design serving as a progenitor in subsequent rounds of directed evolution [52], where all unmodeled latent physical constraints are jointly optimized by mutagenesis and selection.

##### New assays to expand the modelable space

New developments in experimental methods can expand the currently observable protein properties amenable to modeling, often at higher throughput compared to previous methods. Using Tandem Mass Tag (TMT)- multiplexed bottom-up mass spectrometry, Martell *et al*. measured the extent of aggregation after exposure to acidic pH 4 or thermal stress at 50 °C and 75 °C of 18,987 protein domains, all concentration normalized to 10 mg/mL [53]. Ferrari *et al*. quantified residue level energy profiles of protein conformational fluctuations combined across pH 6 and pH 9 for 5,778 protein domains in parallel, distinguishing between all-or-nothing folded to disordered transitions from domains that have partially open intermediates, revealed by differences in hydrogen-deuterium exchange rates for protected or exposed backbone amide hydrogens [54]. Adapting the semi-automated protein production protocol from Qian *et al*. [55], Müntener and Abramson *et al*. scaled NMR to characterize the local fluctuations of 384 designed proteins, with spectrometer time being the bottleneck at 224 per week, with faster data acquisition rates possible at expression volumes scaled up to just 16 mL [56]. Passow *et al*. developed an amplicon/protein bead display method which can express and purify >100,000 proteins in one day, and demonstrated a 3-day protocol to quantify the expression levels and equilibrium dissociation constants of 18,000 protein variants [57]. Taken together, these new experimental techniques could enable incorporating protein energy landscapes, stability at a desired pH or temperature as explicit design objectives [58]. Coupling design with direct measurements of protein dynamics has the potential to directly train generative models on such data, building upon efforts to train on synthetic simulations of conformational ensembles [59].

## Conclusion

Macromolecules exist in a physical world. Despite the unique capabilities of learning systems in integrating information, modeling methods ultimately should respect the underlying physics. As more significant heights are being achieved in designing functional proteins, new solutions will lean more heavily on our ability to model the underlying physical principles. We outline here various strategies in practice today, mainly relying on model architecture and data curation. As lab-in-a-loop and various automation processes become more efficient and mature, data centric design tools will undergo dramatic transformation in the near future.

## Figures and Tables

**Figure 1:**
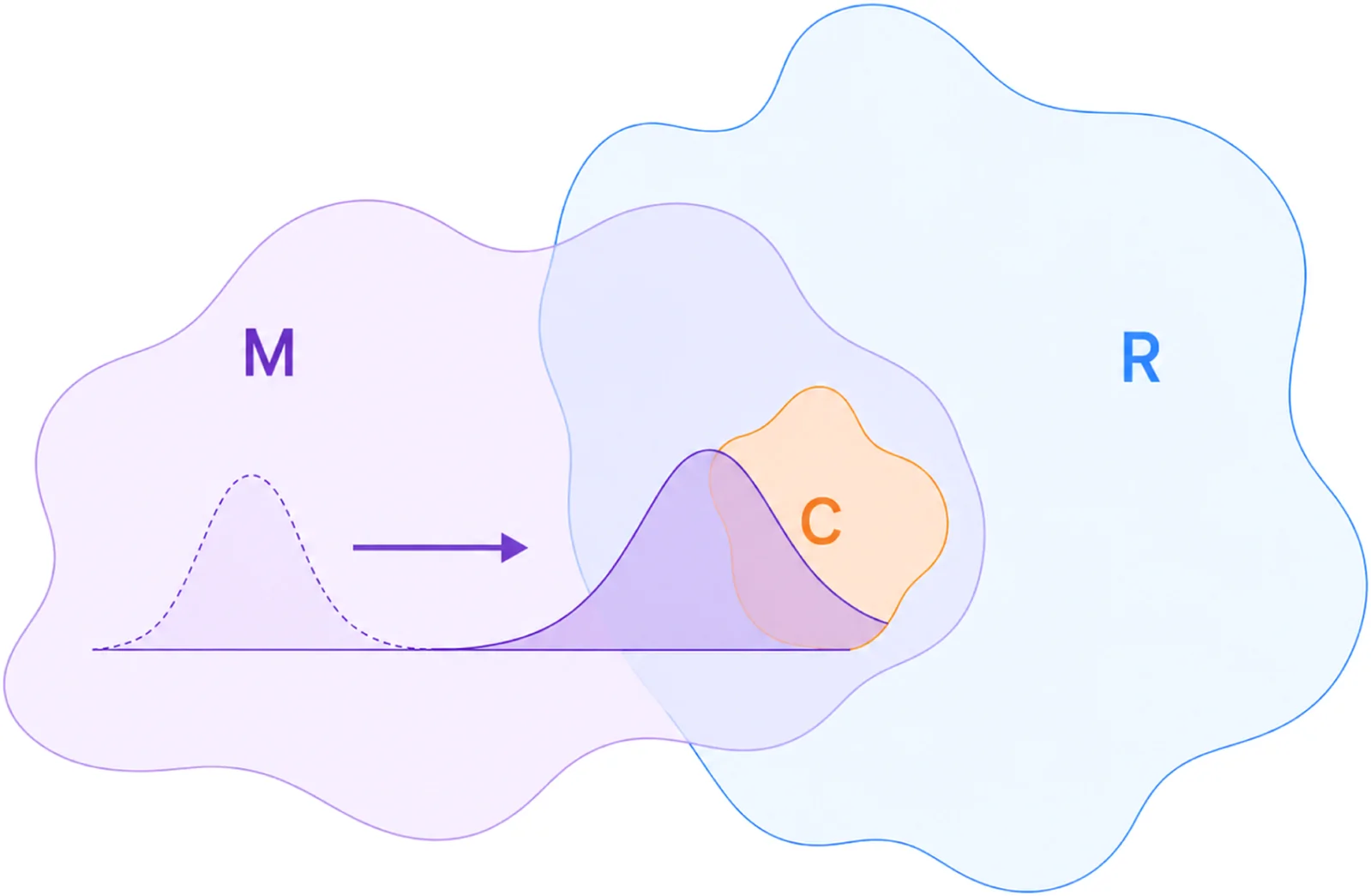
A depiction of the generative design process. **M:** Model distribution. **R:** physically plausible distribution. **C:** design objective distribution. The role of physical terms used in steering or as filters shifts the probability density to increase the proportion of samples that are physically plausible. Without proper specification of such conditions that are necessary and sufficient for an intended designed function, rejection sampling can be highly inefficient. With well-calibrated guidance or a correct set of conditions, the samples can be more enriched for the desired function.

**Figure 2:**
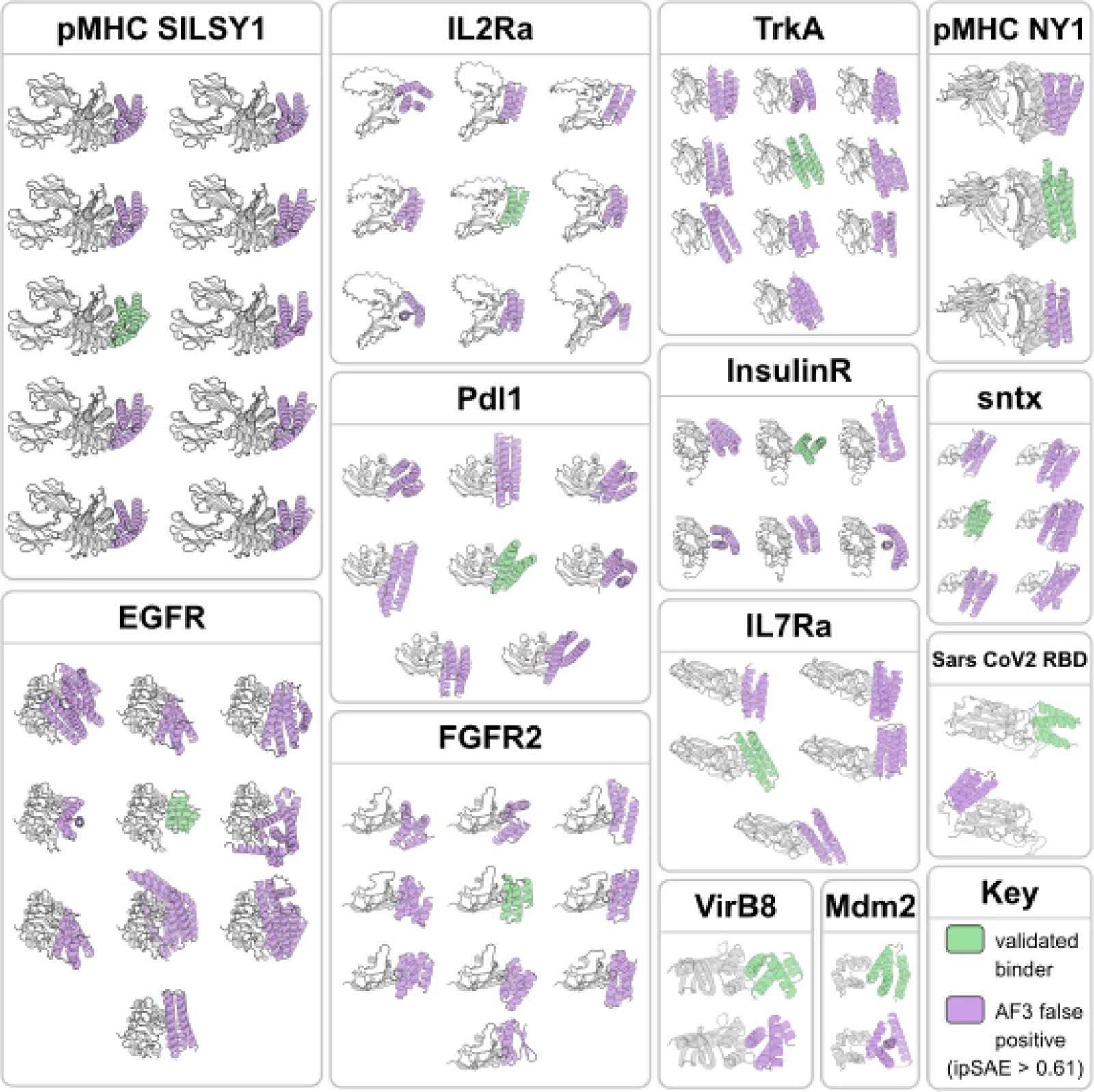
Structure prediction metrics are low precision. Using the AF3 min ipSAE metric threshold of 0.61, a large proportion of designs predicted to be binders (> 0.61) are not binders when tested experimentally. The challenge is to distinguish between the true binders (green) to the target (grey) from the highly plausible designs which fail in experiment (purple). The number of false positives is proportional to the number of true positives given in Overath *et al.* [12].

## Highlights

- Generative models map their latent representations to physically plausible regions of design space
- Desired inference-time distribution is shifted from training distribution but the underlying physics remains constant
- Shaping the model’s physical world view can be done with energy-based losses, architectural regularization, and experimental data feedback
